# Comprehensive Profiling of Cancer-Associated Cells in the Blood of Breast Cancer Patients Undergoing Neoadjuvant Chemotherapy to Predict Pathological Complete Response

**DOI:** 10.3390/bioengineering10040485

**Published:** 2023-04-18

**Authors:** Adity A. Pore, Chathurika S. Dhanasekara, Hunaiz Bin Navaid, Siva A. Vanapalli, Rakhshanda Layeequr Rahman

**Affiliations:** 1 Department of Chemical Engineering, Texas Tech University, Lubbock, TX 79409, USA; adity.pore@ttu.edu (A.A.P.);; 2 Department of Surgery, Texas Tech University Health Sciences Center, Lubbock, TX 79430, USA

**Keywords:** blood biopsy, microfluidics, neoadjuvant chemotherapy, breast cancer, circulating tumor cells (CTC), cancer-associated macrophage like cells (CAMLs)

## Abstract

Neoadjuvant chemotherapy (NAC) can affect pathological complete response (pCR) in breast cancers; the resection that follows identifies patients with residual disease who are then offered second-line therapies. Circulating tumor cells (CTCs) and cancer-associated macrophage-like cells (CAMLs) in the blood can be used as potential biomarkers for predicting pCR before resection. CTCs are of epithelial origin that undergo epithelial-to-mesenchymal transition to become more motile and invasive, thereby leading to invasive mesenchymal cells that seed in distant organs, causing metastasis. Additionally, CAMLs in the blood of cancer patients are reported to either engulf or aid the transport of cancer cells to distant organs. To study these rare cancer-associated cells, we conducted a preliminary study where we collected blood from patients treated with NAC after obtaining their written and informed consent. Blood was collected before, during, and after NAC, and Labyrinth microfluidic technology was used to isolate CTCs and CAMLs. Demographic, tumor marker, and treatment response data were collected. Non-parametric tests were used to compare pCR and non-pCR groups. Univariate and multivariate models were used where CTCs and CAMLs were analyzed for predicting pCR. Sixty-three samples from 21 patients were analyzed. The median(IQR) pre-NAC total and mesenchymal CTC count/5 mL was lower in the pCR vs. non-pCR group [1(3.5) vs. 5(5.75); *p* = 0.096], [0 vs. 2.5(7.5); *p* = 0.084], respectively. The median(IQR) post-NAC CAML count/5 mL was higher in the pCR vs. non-pCR group [15(6) vs. 6(4.5); *p* = 0.004]. The pCR group was more likely to have >10 CAMLs post-NAC vs. non-pCR group [7(100%) vs. 3(21.4%); *p* = 0.001]. In a multivariate logistic regression model predicting pCR, CAML count was positively associated with the log-odds of pCR [OR = 1.49(1.01, 2.18); *p* = 0.041], while CTCs showed a negative trend [Odds Ratio (OR) = 0.44(0.18, 1.06); *p* = 0.068]. In conclusion, increased CAMLs in circulation after treatment combined with lowered CTCs was associated with pCR.

## 1. Introduction

Neoadjuvant chemotherapy (NAC), also known as preoperative chemotherapy, was initially administered in inflammatory or locally advanced breast cancers to reduce the size of inoperable tumors and make them operable [1] or make breast conservation possible in cases that would otherwise require a mastectomy [2,3]. NAC has also been shown to preclude the need for axillary lymph node dissection in node-positive disease by rendering patients node-negative 40% of the time [4]. Therefore, the traditional goal of NAC is to improve surgical outcomes with no impact on survival. However, newer evidence supports the use of second-line treatments in patients with residual disease after NAC improves survival [5,6], thereby expanding the indications of NAC to include patient selection for further therapies that improve survival.

The response to NAC is documented via pathological assessment of the primary tumor bed and regional lymph nodes. The main aim of NAC is to achieve pathological complete response (pCR) [7,8]. pCR is defined as a lack of residual tumor in the primary breast or lymph nodes and is evaluated post-NAC treatment through resection of the primary tumor bed and pathology [9]. Multiple studies, as well as two meta-analyses, have shown that pCR predicts improved long-term outcomes, such as increased disease-free survival (DFS) and overall survival (OS) [10,11,12,13,14,15]. The rate of pCR and choice of second-line therapies are dictated by the phenotypic subtypes of breast cancer [7,11,16,17]. Several studies have found higher rates of pCR in more aggressive tumors, such as human epidermal growth factor receptor 2 (HER2)-positive and triple-negative breast cancers (TNBC) [16,17,18,19,20]. For example, in a study by Asaoka et al. [19], the pCR rates after NAC were 52.9% in HER2-positive/HR-negative breast cancer, 34.2% in TNBC, and 14.7% in HR-positive/HER2-negative breast cancer.

Although pCR is a highly desirable goal, it requires tissue sampling that is only available after the completion of planned NAC since repeated biopsies are impractical. The consequence of this approach is that patients may receive additional doses of toxic therapy that are not effective. Therefore, pragmatic biomarkers that can monitor response and inform adjustments to treatment are needed. An ideal biomarker would: (i) accurately predict pCR such that the need for surgical resection is precluded and (ii) identify drug resistance such that ineffective therapy can be discontinued promptly. Pro-inflammatory cells, such as tumor-infiltrating lymphocytes (TILs), tumor-associated macrophage-like cells (TAMs), and proliferation markers, such as Ki67, have been used as biomarkers for studying response and predicting pCR in the NAC setting [21,22,23]. In a meta-analysis by Mao et al. [23], a higher TIL count in the pretreatment biopsy was correlated with pCR. Likewise, Ki67 was used to evaluate pCR rates in breast cancer patients receiving NAC [24,25]. TILs provide insight into the host immune response to therapy, and Ki67 discerns the oncogenic potential of tumor cells; however, both these markers can be evaluated on tissue specimens that are typically available from the original biopsy or surgical resection. Serial biopsies are impractical for monitoring NAC with such markers because of the invasive nature of the procedure. Besides requiring a tissue biopsy, another drawback of using Ki67 as a biomarker is the significant interobserver variability and lack of consensus on reporting protocol [26]. Similarly, TAMs have been shown to have prognostic utility, and higher counts are associated with higher rates of pCR; however, they pose the same problem of requiring repeated tissue biopsies [22,27,28]. An ideal biomarker would be patient-friendly, accurate, reproducible, and lend itself to uniform interpretation.

Liquid biopsies, being less invasive and easily replicable, harbor a promising avenue for predicting and understanding pCR in the NAC setting. Many proteins, small molecules, and cells circulating the blood have been a subject of research as informants about cancer burden. The oncogenic burden has been studied by identifying circulating tumor cells (CTCs) in the blood of breast cancer patients [29,30,31]. As CTCs are known to be the precursors of metastasis, tracking them across the course of treatment and identifying different phenotypes such as epithelial (E^+^), transitioning (E^+^/M^+^), and mesenchymal (M^+^) cells may provide insights into the prediction of pCR in the NAC setting.

On the other hand, immune cells that interfere with the progress of cancer, such as T-cells, macrophages, neutrophils, natural cells, etc. [32,33,34], can be found in circulation in addition to those that infiltrate the primary tumor [35]. Cancer-associated macrophage-like cells, also known as CAMLs, have been found in the blood of cancer patients [36,37,38,39,40,41]. Studies have suggested that CAMLs are disseminated tumor-associated macrophages (TAMs) that escape the primary tumor microenvironment and enter the circulation. It is unclear if they participate in the phagocytosis of tumor cells or assist in migration during circulation [36,42].

Intuitively, comprehensive profiling of oncogenic and immune cells in the blood will provide insight into the balance between oncogenic potential and host immunity in response to therapy. The changing profile of metastatic potential (CTCs) and host immunity (CAMLs) over the course of treatment might be valuable for predicting pCR in the NAC setting using routine blood draws. Few studies have been done to correlate CTCs with OS and DFS in breast cancer patients undergoing NAC [43,44,45,46,47]. One study isolated E^+^ CTCs before and after NAC and found that triple-negative and HER2-positive cancers were associated with worse OS and DFS if they had ≥2 CTCs/7.5 mL of blood before NAC [43]. They also found that the patients who achieved pCR and had no CTCs before NAC exhibited better OS and DFS than those with residual cancer and CTCs present before NAC. However, a drop in CTCs on NAC has not been consistently correlated with pCR increasing the possibility of inadequate profiling of CTC phenotypes and host immune cells, thereby limiting the information obtained for accurate prediction of metastasis and survival [48].

In this preliminary longitudinal study, we temporally interrogated circulating blood to evaluate the CTCs and CAMLs (circulating counterparts of TAMs) and document their association with treatment outcomes in patients undergoing NAC using liquid biopsy and the label-free Labyrinth microfluidic technology [49,50,51,52]. Previously, label-free microfilter technology CellSieve™ was shown to isolate CTCs and CAMLs from the blood of breast, prostate, and pancreatic cancer patients [36]. However, there is a lack of understanding of how different cancer-associated cells, such as CTCs (all phenotypes) and CAMLs, change in circulation as NAC progresses. This study provides pilot data to prove the concept of the role of comprehensive profiling of cancer-associated cells in the blood in patients undergoing NAC at different time points.

## 2. Methods

**Figure 1:**
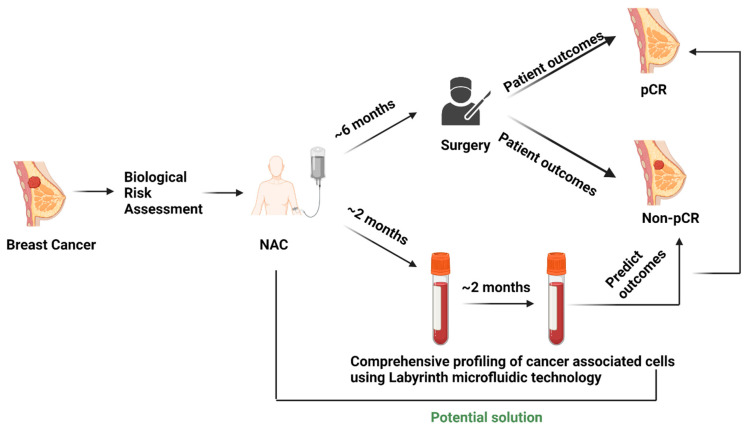
Current scenario of surgery-based patient outcome identification in the NAC setting and testing liquid biopsy as a potential predictive biomarker.

The experimental protocol (Figure 1) was approved by the Institutional Review Board (Protocol # L19-043) of Texas Tech University Health Sciences Center (TTUHSC), Lubbock. The multidisciplinary breast cancer team evaluated all newly diagnosed breast cancer patients. The treatment strategy was planned after the tumor board discussion. Patients selected for NAC with an intention to cure who consented to participate were enrolled in the study.

### 2.1. Sample Collection

A total of 5 mL of whole blood was collected from patients in K2 EDTA tubes (BD Vacutainer tubes, Franklin Lakes, NJ, USA) before starting treatment (pre-), during therapy (mid-), and after the completion of NAC (post-).

### 2.2. Sample Pre-Processing and CTC Isolation

The red blood cells (RBCs) were depleted from 5 mL of whole blood per sample using a Ficoll density gradient. Peripheral blood mononuclear cells (PBMCs) were collected after RBC depletion and diluted 5x before processing in the Labyrinth microfluidic technology [49]. The Labyrinth technology uses inertial focusing to separate cancer-associated cells based on size and deformability. The preparation of the Labyrinth microfluidic chip and the sample processing was carried out as described by Lin et al. [49].

### 2.3. Immunostaining for Cell Identification

The enriched CTC sample was collected from the Labyrinth chip and immunostained for cell identification and phenotyping. Collected cells were deposited on a coated glass slide using Cytospin (Epredia, Kalamazoo, MI, USA). These cells were further fixed using 4% paraformaldehyde (Thermo Fisher Scientific, Waltham, MA, USA) and permeabilized using 0.2% Triton X-100 (Sigma Aldrich, St. Louis, MO, USA) for 3 min. The cells were washed 3 times for 5 min each using phosphate buffer saline (PBS Gibco, Gaithersburg, MD), followed by a blocking step using 10% normal goat serum (Thermo Fisher Scientific, Waltham, MA, USA) for 30 min. After blocking, the cells were incubated in a humidified chamber with a primary antibody mix for 8 h at 4 °C, followed by a 3× PBS wash. The primary antibody mix consisted of mouse anti-human CD45 IgG2 (Bio-Rad, Hercules, CA, USA), mouse anti-human PanCK IgG1 (Bio-Rad, Hercules, CA, USA), and rabbit anti-human Vimentin (Abcam, Waltham, MA, USA). After the primary antibody incubation and wash steps, the cells were incubated with a mix of secondary antibodies (Thermo Fisher Scientific, Waltham, MA, USA) for 1.5 h. The secondary antibody mix comprised goat anti-mouse IgG2 AF 488, goat anti-mouse IgG1 AF 546, and goat anti-rabbit AF 647. Lastly, the slide was washed 3x with PBS and mounted with a cover glass (Richard-Allan Scientific, Kalamazoo, MI, USA) using Prolong Gold Antifade with DAPI (Thermo Fisher Scientific, Waltham, MA, USA). Unless otherwise specified, all incubation times and washing steps were carried out at room temperature.

### 2.4. Imaging for Cell Enumeration

**Figure 2:**
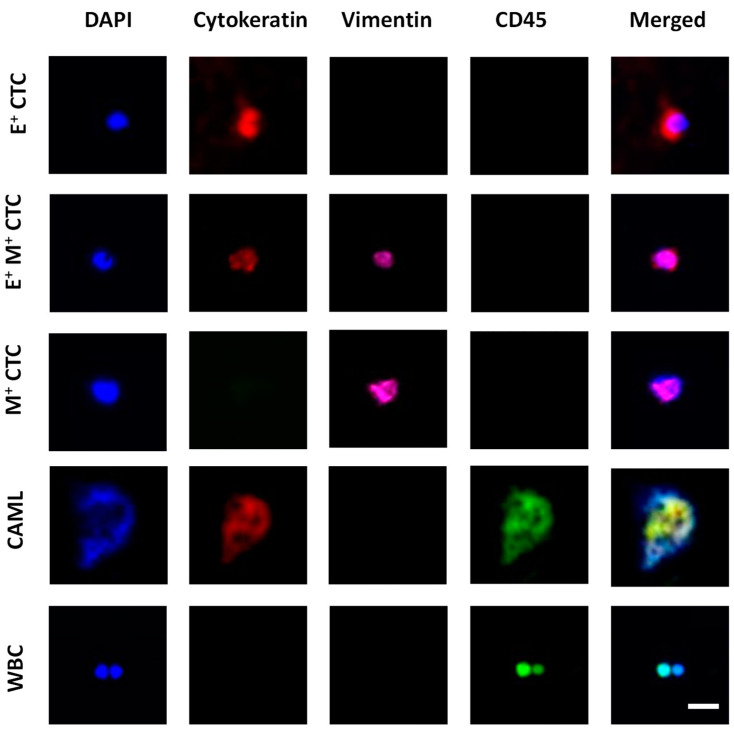
Image panel showing the different tumor-associated cells characterized in blood. Epithelial CTCs (E^+^ CTCs) stained positive for DAPI (blue) and Cytokeratin (red). Transitioning CTCs (E^+^M^+^ CTCs) stained positive for DAPI, Cytokeratin, and Vimentin (pink). Mesenchymal CTC (M^+^ CTC) stained positive for DAPI and Vimentin. CAML stained positive for DAPI, Cytokeratin, and CD45 (green). White blood cells (WBCs) stained positive for DAPI and CD45. Scale bar is 20 μm.

Fluorescent images of the immunostained samples were acquired using a Hamamatsu ImageEM-CCD digital camera (512 × 512 pixels, Bridgewater, NJ, USA) mounted to an Olympus IX81 microscope (Waltham, MA, USA). The microscope was equipped with a motorized stage controlled by Slidebook 6.1 software (3i Intelligent Imaging Innovations Inc., Denver, CO, USA). Four-filter fluorescent images were acquired under DAPI, FITC, TRITC, and Cy5 filters with exposure times ranging from 20–100 ms. The acquired images were analyzed using Slidebook Reader (3i Intelligent Imaging Innovations Inc., Denver, CO, USA). Figure 2 shows an example set of images for cell identification.

### 2.5. Statistical Analysis

Data were imported to R statistical software and the distributions were examined visually. A Shapiro-Wilk test of normality was conducted to assess the normality. The Shapiro-Wilk normality test was significantly different (*p* < 0.05), indicating that data distribution was not normal. Thus, non-parametric tests were conducted. Medians and interquartile ranges were used to summarize variables. A Wilcoxon rank-sum test was used to compare pCR and non-pCR group medians separately for all three time points. A two-sample Wilcoxon signed-rank test compared the pre- vs. post-NAC group medians.

The CTC categories were created based on the threshold of having ≥1 CTCs/5 mL or not [53]. Similarly, categories for CAMLs were formed based on whether there were ≥10 CAMLs/5 mL isolated. A Fisher’s exact test was used for comparisons of pCR with non-pCR group. Paired prevalence of CTCs and CAMLs were compared between pre- –and post-NAC samples using a McNemar Exact test. Outlier values of CAML and CTC counts were replaced by winsorizing data at the 5th and 95th percentiles (values of the 5th percentile and below or 95th percentile and above were set to this value) [54]. Models were constructed after winsorizing data. The association between pCR and CAML, CTC, and combination were examined in three separate logistic regression models. The three models’ fit indices (i.e., Akaike Information Criterion: AIC and likelihood ratios) were compared after adjusting for model parsimony. Model comparisons were made using the ‘AICcmodavg’ package in R. All statistical analyses were conducted using R statistical software (version 4.1.3) [55]. A *p*-value of less than 0.05 was considered significant.

## 3. Results

### 3.1. Baseline Patient Characteristics and Treatment Outcomes

Between September 2020 and February 2022, 21 breast cancer patients were enrolled in the study. The median age was 53 (27) years; all patients received complete NAC as planned. The clinicopathological characteristics of the patients and response to NAC are shown in Table 1. A total of 10 (47.6%) had stage II disease, while 10 (47.6%) had stage III disease at presentation. Out of 21 patients, 7 (33.3%) patients had a triple-negative tumor, 6 (28.6%) patients were HER2+ and 8 (38.1%) patients were HR+/HER2−. After NAC, 2 (9.5%) triple-negative cancer patients, 3 (14.3%) HER2+ cancer patients, and 2 (9.5%) HR+/HER2− cancer patients achieved pCR.

### 3.2. Distribution of CTC Phenotypes in Peripheral Blood of Breast Cancer Patients Undergoing NAC

Each of the 21 patients had three blood draws (pre-, mid-, and post-NAC) for 63 samples.

The distribution of CTC phenotypes between the pCR and non-pCR groups, enumerated from each draw, is shown in Table 2. Whereas the median total number of CTCs before NAC was lower [median = 1 (IQR = 3.5)] in the pCR group compared with the non-pCR group [median = 5 (IQR = 5.750)], it did not reach statistical significance (*p* = 0.096). However, patients with higher mesenchymal CTC counts before therapy were less likely to have pCR [2.5 (IQR = 7.5) vs. zero; *p* = 0.084]. There was a trend of patients with higher mesenchymal CTCs in the non-pCR group at all time points, albeit this difference was not statistically significant. No significant differences in median E^+^ CTCs were observed at any time point between pCR and non-pCR groups and pre- vs. post-NAC comparisons.

There was no significant difference in the total number of CTCs before and after NAC in both the treatment outcome groups. Overall, mesenchymal CTCs were the major contributors to the total number of CTCs in both patient outcome groups in all three draws.

### 3.3. Distribution of CAMLs in Peripheral Blood of Breast Cancer Patients Undergoing NAC

Table 3 shows the distribution of CAMLs in the pre-, mid-, and post-NAC blood samples in the pCR and non-pCR groups.

Patients in the pCR group had fewer CAMLs compared to the non-pCR group before NAC, albeit this difference was not statistically significant [median = 3 (IQR = 3) vs. median = 7 (IQR = 7); *p* = 0.092]. However, this pattern reversed after completion of NAC [median = 15 (IQR = 6) vs. median = 6 (IQR = 4.5); *p* = 0.004]. Interestingly, we found a significantly high number of CAMLs in the pCR group as compared to the non-pCR group (Δ = 9 CAMLs/5 mL, W = 10.5, *p* = 0.004).

We found that the difference in the numbers of CAMLs before and after NAC in the pCR group was statistically significant (Δ = 12 CAMLs/5 mL, V = 0, *p* = 0.022). However, no difference was found between the pre- and post-NAC CAML counts in the non-pCR group. Overall, the CAMLs showed an increasing trend in the pCR group as the treatment progressed.

### 3.4. Categorical Analysis of CTC Phenotypes and CAMLs in the Blood of Breast Cancer Patients Undergoing NAC

To understand the relationship between the CTC load and treatment outcomes, the pCR and non-pCR patients were categorized into cohorts of 0 CTCs or ≥1 CTCs/5 mL. When considering E^+^ CTCs categories, there were no significant differences in E^+^ CTCs categories at any time point (Table 4). Two patients from the pCR group (28.6%) and 8 (57.1%) patients from the non-pCR group had ≥1 E^+^ CTCs in their pre-NAC samples, and 3 (42.9%) patients from the pCR group and 6 (42.9%) patients from the non-pCR group had ≥1 E^+^ CTCs in their post-NAC sample.

There was no significant difference in the mesenchymal CTC counts between pCR and non-pCR groups across three time points, as shown in Table 5. However, the number of patients with ≥1 mesenchymal CTC count rose significantly among the non-pCR patients from treatment naïve to post-NAC status [n = 10 (71.4%) vs. n = 13 (92.9%); *p* = 0.012].

There was no difference between the pCR and non-pCR groups when comparing the total CTC counts, albeit the trend suggested increasing counts among non-pCR patients at each time point (Table 6). Interestingly, the number of patients with ≥1 total CTC count rose significantly among the non-pCR patients from treatment naïve to post-NAC status [n = 11 (78.6%) vs. n = 13 (92.9%); *p* = 0.001].

CAML response was assessed using a cutoff of 10 cells/5 mL. All patients in the pCR group (n = 7, 33.33%) flipped from a treatment naïve status of ≤10 CAML count to a post-NAC status of >10 CAML count (Table 7). This is an intriguing observation, although the significance cannot be calculated due to the paired nature of data required by the McNemar exact test. There were significant differences between the pCR and non-pCR groups in CAML counts at the mid- and post-NAC time points (*p* = 0.18 and 0.001, respectively), albeit the direction of difference reversed from the mid- to the post-NAC time point.

### 3.5. Effect of CTCs and CAMLs on pCR

**Figure 3:**
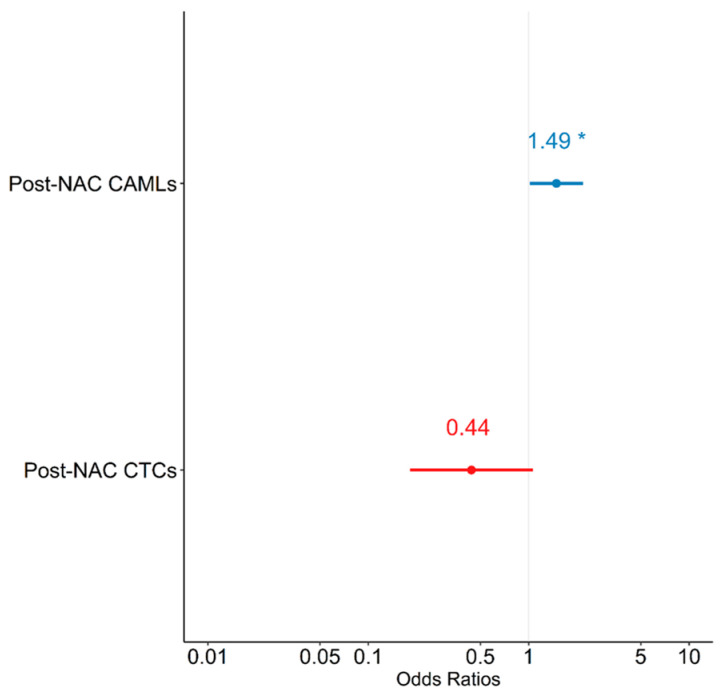
Forest plot depicting multivariate model predicting pathological complete response (pCR). NAC = neoadjuvant chemotherapy; CAMLs = cancer-associated macrophage-like cells; CTCs = circulating tumor cells. * *p* < 0.05.

Logistic regression models were constructed to evaluate the relationship between pCR and CTCs and CAMLs. A univariate analysis did not show any significant associations between post-NAC CAML or post-NAC CTCs and pCR—except for a trend in increasing post-NAC CAMLs, which was associated with an increase in log odds of achieving pCR (*p* = 0.079) (Table 8). In a multivariate logistic regression model predicting pCR, when controlling for CTCs, there was a unit increase in post-NAC CAMLs; furthermore, the log odds of achieving pCR were observed to increase by 0.399 (Table 8). Similarly, when controlling for CAMLs there was a unit increase in post-NAC CTCs, wherein the log odds of achieving pCR were observed to decrease by 0.823, which did not reach statistical significance. The multivariate model suggests that patients with increased levels of post-NAC CAMLs were at increased odds of achieving pCR [OR = 1.490 (1.017, 2.182); *p* = 0.042], while CTCs showed a trend in negatively predicting pCR [OR = 0.439 (0.182, 1.062); *p* = 0.068] (Figure 3). Therefore, when controlling for each other, increased levels of CAML and decreased levels of CTCs were associated with increased odds of pCR.

The model that included the combination of CAMLs and CTCs (i.e., model 3) outperformed the two univariate models (i.e., models 1 and 2) based on likelihood ratio (*p* < 0.001). Similarly, the AIC of model 3 (AIC = 19.374) was significantly lower than the AIC statistics of model 1 and 2 (AIC = 25.990, *p* = 0.003 and AIC = 30.082, *p* < 0.001). These goodness-of-fit criteria indicated that the combination of post-NAC CAML and CTC counts is superior to either CAML or CTC counts alone when predicting pCR, thereby carrying 94% of the cumulative model weight.

## 4. Discussion

Neoadjuvant therapy is administered to patients before the surgical removal of the tumor in order to shrink the tumor and make it operable, and, more recently, to identify non-responders for second-line therapies. However, there is no clinically practical non-invasive methodology to track the response of treatment in patients undergoing NAC [56,57] until the completion of a predetermined regimen. The translational value of liquid biopsies lies in its capability for enabling real-time monitoring of the treatment progress of NAC. Real-time monitoring using liquid biopsies can help curtail the toxicity of non-effective chemotherapeutic drugs in patients and can help clinicians with decision-making regarding switching to better-targeted therapies in a timely manner [58,59]. For example, in an ongoing I-SPY 2 clinical trial to evaluate the efficacy of chemotherapeutic drugs that has been running for over a decade, the effectiveness of a drug is evaluated after long-term follow-ups with patients by conducting serial biopsies and MRIs [60,61,62]. In a long-term trial such as I-SPY 2, comprehensive liquid biopsies can be used for real-time monitoring of treatment in patients, which could potentially reduce the time taken to deem the effectiveness of the drug based on the genomic profile of the tumor in patients.

Several studies have been done in a similar context with CTCs and NAC in breast cancer patients [43,45,47,63,64,65]. However, most of these studies have used label-based techniques to isolate CTCs [63,64,65]. The major drawback of label-based isolation techniques is that different phenotypes of CTCs cannot be captured and enumerated, which precludes the evaluation of patient outcomes in a comprehensive context [66,67,68]. A few studies that used label-free isolation of CTCs did not enumerate each phenotype to identify their independent role. For example, Ni et al. [46] isolated CTCs using the CanPatrol [69] technology and classified patients as CTC-positive or CTC-negative. This approach potentially provided the total CTC load; however, the independent effect of epithelial versus mesenchymal CTCs could not be studied. In addition, it is unclear if researchers encountered CAMLs with the CanPatrol technology.

In another study done by O’Toole et al. [70], CTCs were enumerated to assess the correlation with pCR. Blood samples from 26 patients were analyzed pre- and post-NAC using the label-free ScreenCell [71] technology. They reported a 19.2% pCR rate, which is lower than what is typically seen in current-day practice [72]. The low rate of pCR in this study is most likely due to 57.7% of the group being represented by luminal A cancers, which are least likely to respond. In addition, they used 5 CTCs/3 mL of whole blood as a cutoff for categorization and reported no significant correlation with pCR. The authors intend to re-analyze their data at a longer follow-up for clinical outcomes. However, we believe that a lack of information on CTCs in transition to mesenchymal cells and cells representing host immune response may continue to confound the conclusions.

Adams et al. [36] considered the host immune response by identifying CAMLs in the blood of early and late-stage breast, pancreatic, and prostate cancer patients. They reported that CAMLs were present in high numbers in cancer patients undergoing chemotherapy (29 CAMLs/7.5 mL) compared to treatment naïve patients (4 CAMLs/7.5 mL). This phenomenon is consistent with our finding of increasing CAML counts, particularly in patients responding well to systemic therapy. Whereas this study focused on host immune response, the CTCs’ oncogenic potential was not addressed.

As CAMLs are the disseminated counterparts of TAMs, they can further be investigated based on their phenotypic polarity of switching from M1 and M2 and vice versa [36,42,73,74,75,76,77,78]. M1 TAMs, also known as pro-inflammatory TAMs, have been known to fight against cancer and are associated with a positive prognosis, whereas M2 TAMs are known to promote angiogenesis by releasing vascular epithelial growth factors (VEGFs) and are known to be associated with negative patient outcomes [79]. In a study by Petrillo et al. [78], locally advanced cervical cancer patients undergoing chemoradiation showed pCR (no residual tumor) when the M1/M2 ratio was high. Additionally, the women with a high M1/M2 ratio also showed a longer disease-free and overall survival as compared to the women with a lower M1/M2 ratio [78]. Therefore, it would be important to investigate CAMLs based on M1 and M2 phenotypes to better understand the treatment outcomes in patients undergoing NAC.

We believe that cancer metastasis and response to systemic therapy involve complex processes and must be studied in a broad context. To the best of our knowledge, this is the first report on the comprehensive profiling of cancer-associated cells in the circulation of cancer patients that represent both the oncogenic and immune response processes. Combining the oncogenic profile of tumor cells and the immune profile of host cells is essential in understanding the depth of clinical outcomes in breast cancer patients undergoing NAC. This paper demonstrates the interplay between CTC phenotypes and CAMLs in response to NAC and their potential role in achieving pCR. However, our sample size is small, limiting us from developing a reliable model for the prediction of pCR. We intend to continue to enroll more patients to allow for robust analysis of the CTC–CAML interaction, particularly in the context-specific genomic profiles of breast cancer since they respond differently to NAC.

The limitation of our study lies in the small sample size for the investigation of CTC–CAML interactions. A multi-institutional study with a large sample size would clarify this concept more robustly; however, the logistics of collecting fresh blood samples at convenient times for patients and immediately transporting them for processing precluded this approach at this time. The blood samples were immediately processed post-draw to obtain accurate CTC and CAML counts in the blood of the patients. Our group continues to work on technological advancement for processing these samples centrally without compromising the accuracy of detection to allow for the enrollment of patients from several distant sites. In the future, our goal is to proliferate this pipeline and conduct a multi-institutional trial where several patients are enrolled, and a larger sample size can validate the findings of our study. Moreover, a larger sample size will not only enable us to study this unique CTC–CAML interaction and patient outcomes, but also enhance the precision of prediction phenomena in patient subgroups, such as pre- vs. post-menopausal women, genomic (as opposed to phenotypic) profiles of tumors, etc. The current study did not differentiate between M1 and M2 CAMLs; a larger sample will also allow for that discrimination, as well as how this difference adds precision to the score for predicting the response to NAC.

## 5. Conclusions

Our preliminary investigation shows that the best prediction of pCR is expected to arise from decreasing CTC counts (particularly mesenchymal CTCs) with a simultaneous increase in host immune response. The CTC–CAML interaction is likely to become increasingly relevant as newer immunotherapies arise. In summary, the Labyrinth microfluidic technology offers a promising venue for comprehensive profiling of cancer-associated cells that can be leveraged to answer several relevant clinical questions as we move towards precision medicine.

## Figures and Tables

**Table 1:** Patient characteristics.

	N (%)	pCR	Non-pCR	*p*-Value
All patients	21 (100.0)	7	14	0.189
Age				
≤50	8 (38.1)	2	6	0.289
<50	13 (61.9)	5	8	0.581
Tumor size				
cT1	2 (9.5)	1	1	1.500
cT2	7 (33.3)	2	5	0.453
cT3	9 (42.9)	4	5	1.000
cT4 a-c	1 (4.8)	0	1	1.000
cT4d	2 (9.5)	0	2	0.500
Node				
cN0	4 (19.0)	2	2	1.375
cN1-3	17 (81.0)	5	12	0.143
Tumor Grade				
II	9 (42.9)	3	6	0.508
III	12 (57.1)	4	8	0.376
Stage				
I	1 (4.8)	0	1	1.000
II	10 (47.6)	4	6	0.754
III	10 (47.6)	3	7	0.344
Ki67				
≤20	1 (4.8)	0	1	1.000
<20	20 (95.2)	7	13	0.263
Tumor Subtype				
Triple Negative	7 (33.3)	2	5	0.453
HER2+	6 (28.6)	3	3	1.313
HR+/HER2−	8 (38.1)	2	6	0.289

HR+/HER2− contain ER+/PR+, ER+/PR− and ER−/PR+ subgroups. *p* values were determined by Fisher’s exact test. HR = hormone receptor, ER = estrogen receptor, PR = progesterone receptor, HER2 = human epidermal growth factor receptor 2, pCR = pathological complete response.

**Table 2:** Summary of CTC phenotypes based on treatment outcomes in pre-, mid-, and post-draws of patients undergoing NAC.

Draw	Phenotype	CTCs/5 mL Median (IQR)		*p*-Value ^ɣ^
		pCR (n = 7)	Non-pCR (n = 14)	
Pre-NAC	E^+^	0 (0.5)	1 (1)	0.368
	M^+^	0 (1)	2.5 (7.5)	0.084
	E^+^M^+^	0 (0)	0 (1)	0.425
	Total	1 (3.5)	5 (7.5)	0.096
Mid-NAC	E^+^	0 (0)	1.5 (3.5)	0.098
	M^+^	1 (6)	2 (2.75)	0.544
	E^+^M^+^	0 (1)	0.5 (1)	0.806
	Total	1 (6)	5 (7.5)	0.133
Post-NAC	E^+^	0 (1.5)	0 (1)	0.934
	M^+^	3 (1)	3.5 (6)	0.733
	E^+^M^+^	0 (1)	0.5 (1)	0.805
	Total	4 (1)	6 (8)	0.524
Pre-Post difference in Total CTCs ^†^		0.235	0.552	

^ɣ^ Wilcoxon rank-sum test; ^†^ Wilcoxon signed-rank test; NAC = neoadjuvant chemotherapy, pCR = pathological complete response, CTCs = circulating tumor cells, E^+^ = epithelial, M^+^ = mesenchymal, E^+^M^+^ = epithelial to mesenchymal.

**Table 3:** Distribution of CAMLs based on treatment outcomes in pre-, mid-, and post-draws of patients undergoing NAC.

Draw	CAMLs/5 mL Median (IQR)		*p*-Value ^ɣ^
	pCR (n = 7)	Non-pCR (n = 14)	
Pre-NAC	3 (3)	7 (7)	0.092
Mid-NAC	8 (1.5)	11 (32.75)	0.153
Post-NAC	15 (6)	6 (4.5)	0.004
Pre-Post difference in CAMLs ^†^	0.022	0.833	

^ɣ^ Wilcoxon rank-sum test; ^†^ Wilcoxon signed-rank test. NAC = neoadjuvant chemotherapy, pCR = pathological complete response, CAMLs = cancer-associated macrophage-like cells.

**Table 4:** Categorization of patients based on E^+^ CTC load and treatment outcomes across pre-, mid-, and post-NAC draws.

Draw	E^+^ CTCs/5 mL	No. of Patients (%)		*p*-Value ^ɣ^
		pCR (n = 7)	Non-pCR (n = 14)	
Pre-NAC	0	5 (71.4)	6 (42.9)	0.362
	≥1	2 (28.6)	8 (57.1)	
Mid-NAC	0	6 (85.7)	6 (42.9)	0.159
	≥1	1 (14.3)	8 (57.1)	
Post-NAC	0	4 (57.1)	8 (57.1)	1.0
	≥1	3 (42.9)	6 (42.9)	
Pre-Post ^†^		0.688	1.0	

^ɣ^ Fisher’s exact test; ^†^ McNemar exact test; NAC = neoadjuvant chemotherapy, pCR = pathological complete response, CTCs = circulating tumor cells, E^+^ = epithelial.

**Table 5:** Categorization of patients based on M^+^ CTC load and treatment outcomes across pre-, mid-, and post-NAC draws.

Draw	M^+^ CTCs/5 mL	No. of Patients (%)		*p*-Value ^ɣ^
		pCR (n = 7)	Non-pCR (n = 14)	
Pre-NAC	0	4 (57.1)	4 (28.6)	0.346
	≥1	3 (42.9)	10 (71.4)	
Mid-NAC	0	3 (42.9)	3 (21.4)	0.354
	≥1	4 (57.1)	11 (78.6)	
Post-NAC	0	0 (0)	1 (7.1)	1.0
	≥1	7 (100)	13 (92.9)	
Pre-Post ^†^		0.25	0.012	

^ɣ^ Fisher’s exact test; ^†^ McNemar exact test; NAC = neoadjuvant chemotherapy, pCR = pathological complete response, CTCs = circulating tumor cells, M^+^ = mesenchymal.

**Table 6:** Categorization of patients based on total CTC load and treatment outcomes across pre-, mid-, and post-NAC draws.

Draw	Total CTCs/5 mL	No. of Patients (%)		*p*-Value ^ɣ^
		pCR (n = 7)	Non-pCR (n = 14)	
Pre-NAC	0	3 (42.9)	3 (21.4)	0.354
	≥1	4 (57.1)	11 (78.6)	
Mid-NAC	0	2 (28.6)	0 (0)	0.1
	≥1	5 (71.4)	14 (100)	
Post-NAC	0	0 (0)	0 (0)	0.127
	≥1	7 (100)	14 (100)	
Pre-Post ^†^		0.125	0.001	

^ɣ^ Fisher’s exact test; ^†^ McNemar exact test; NAC = neoadjuvant chemotherapy, pCR = pathological complete response, CTCs = circulating tumor cells.

**Table 7:** Categorization of patients based on CAML load and treatment outcomes across pre-, mid-, and post-NAC draws.

Draw	CAMLs/5 mL	No. of Patients (%)		*p*-Value ^ɣ^
		pCR (n = 7)	Non-pCR (n = 14)	
Pre-NAC	≤10	7 (100)	10 (71.4)	0.255
	>10	0 (0)	4 (28.6)	
Mid-NAC	≤10	7 (100)	6 (42.9)	0.018
	>10	0 (0)	8 (57.1)	
Post-NAC	≤10	0 (0)	11 (78.6)	0.001
	>10	7 (100)	3 (21.4)	
Pre-Post ^†^		Cannot calculate	0.119	

^ɣ^ Fisher’s exact test; ^†^ McNemar exact test; NAC = neoadjuvant chemotherapy, pCR = pathological complete response, CAMLs = cancer-associated macrophage-like cells.

**Table 8:** Univariate and multivariate logistic regression models predicting pathological complete response (pCR).

Independent Variables	Betas	SEs	Zs	OR [95% CI]	*p*-Value
Univariate Model—Model 1					
Intercept	−2.159	0.959	−2.251	0.115 [0.018, 0.757]	0.024
Post-NAC CAMLs	0.126	0.072	1.756	1.134 [0.986, 1.305]	0.079
Univariate Model—Model 2					
Intercept	−0.174	0.787	−0.221	0.84 [0.18, 3.93]	0.825
Post-NAC CTCs	−0.088	0.113	−0.776	0.916 [0.734, 1.143]	0.438
Multivariate Model—Model 3					
Intercept	−1.495	1.202	−1.244	0.224 [0.021, 2.366]	0.214
Post-NAC CAMLs	0.399	0.195	2.048	1.49 [1.017, 2.182]	0.041
Post-NAC CTCs	−0.823	0.451	−1.826	0.439 [0.182, 1.062]	0.068

NAC = neoadjuvant chemotherapy; CAMLs = cancer-associated macrophage-like cells; CTCs = circulating tumor cells; SE = standard error; Z = Z statistic, OR = odds ratio; CI = confidence interval.

## Data Availability

The data presented in this study will be made available on request from the corresponding author.
